# Novel Porcine Model of Coronary Dissection Reveals the Impact of Impella on Dissected Coronary Arterial Hemodynamics

**DOI:** 10.3389/fcvm.2020.00162

**Published:** 2020-09-15

**Authors:** Taro Kariya, Kelly P. Yamada, Olympia Bikou, Serena Tharakan, Satoshi Miyashita, Kiyotake Ishikawa

**Affiliations:** Cardiovascular Research Center, Icahn School of Medicine at Mount Sinai, New York, NY, United States

**Keywords:** coronary arterial dissection, spontaneous coronary artery dissection, impella, large animal, LV unloading, coronary angiography, intimal flap

## Abstract

**Background:** Coronary artery dissection (CAD) sometimes accompanies unstable hemodynamics and requires mechanical cardiac support. Meanwhile, mechanical cardiac support may influence coronary hemodynamics in CAD. No study has examined the impact of Impella left ventricular (LV) support on CAD.

**Materials and Methods:** CAD was induced in eight Yorkshire pigs by injuring the left anterior descending artery (LAD) using a 0.018-in. stiff guidewire and/or deep engagement of a blunt-cut coronary guiding catheter. After the creation of CAD, hemodynamic parameters, coronary pressure, and flow as well as coronary angiograms were acquired before and after maximum LV support using the Impella CP.

**Result:** CADs with a large flap were successfully created by deep engagement of a blunt-tip guiding catheter with forceful contrast injection. One animal (#8) exhibited thrombolysis in myocardial infarction (TIMI)-1 flow, while the others (animals #1–#7) showed TIMI-2/3 flow. In TIMI-2/3 animals, maximal Impella support increased mean coronary pressure (108.4 ± 22.5 to 124.7 ± 28.0 mmHg, *P* < 0.001) with unchanged mean coronary flow velocity (63.50 ± 28.66 to 48.32 ± 13.30 cm/s, *P* = 0.17) of the LAD distal to the dissection. The LV end-diastolic pressure (20.6 ± 6.6 vs. 12.0 ± 3.4 mmHg, P = 0.032), LV end-diastolic volume (127 ± 32 vs. 97 ± 26 ml, *P* = 0.015), stroke volume (68 ± 16 vs. 48 ± 14 ml, *P* = 0.003), stroke work (5,744 ± 1,866 vs. 4,424 ± 1,650 mmHg·ml, *P* = 0.003), and heart rate (71.4 ± 6.6 vs. 64.9 ± 9.3/min, *P* = 0.014) were all significantly reduced by Impella support, indicating effective unloading of the LV. In the TIMI-1 animal (animal #8), maximal Impella support resulted in further delay in angiographic coronary flow and reduced distal coronary pressure (22.9–17.1 mmHg), together with increased false-lumen pressure.

**Conclusion:** Impella support effectively unloaded the LV and maintained the hemodynamics in a novel porcine model of CAD. Coronary pressure distal to the dissection was increased in TIMI-2/3 animals after Impella support but decreased in the animal with initial TIMI-1 flow.

## Introduction

Coronary artery dissection (CAD) is a rare but challenging condition that can cause hemodynamic compromise. The majority of CADs are traumatic or iatrogenic in nature, the latter occurring as a sequela of percutaneous coronary intervention (PCI) (1, 2). Iatrogenic coronary dissection is often associated with subsequent myocardial ischemia and can also lead to cardiogenic shock, potentially requiring mechanical circulatory support (1, 2). Non-traumatic, non-iatrogenic, and non-atherosclerotic CAD is termed spontaneous CAD (SCAD). SCAD is difficult to treat, as this condition leads to friable coronary arteries, making them susceptible to additional iatrogenic dissection or extension of dissections during coronary angiography and PCI. PCI or coronary artery bypass grafting (CABG) is considered in the presence of ongoing ischemia, left main artery dissection, or hemodynamic instability in SCAD patients (3). Stabilizing patient hemodynamics and relieving the ischemic burden are of paramount importance for all types of CAD that accompany ischemia.

The use of mechanical circulatory support devices in the setting of high-risk PCI and cardiogenic shock is on the rise (4). These devices can be rapidly deployed in the emergency department or catheterization suite to stabilize patient hemodynamics and unload the left ventricle (LV). Specifically, the Impella heart pump (Abiomed, Danvers, MA) is a catheter-based transaortic LV microaxial assist device that has been gaining in popularity and utilization (5). Owing to its position across the aortic valve, the Impella augments cardiac output and mean arterial pressure while simultaneously decreasing the workload of the heart (6) and increasing coronary blood flow into the infarct zone of the LV after myocardial infarction (7). Although this pleiotropic ability of the Impella device to stabilize hemodynamics and attenuate ischemic burden makes it a therapeutic candidate for CAD patients, no study has investigated the impact of Impella in a setting of dissected coronary artery. Moreover, there are no adequate animal models for testing for the effect of Impella in CAD.

Here, we developed a novel porcine model of CAD and examined the impact of Impella on LV and coronary hemodynamics. We hypothesized that Impella LV support not only unloads the LV but also changes coronary pressure and flow distal to the dissected coronary artery.

## Materials and Methods

### Study Protocol

**Figure 1:**
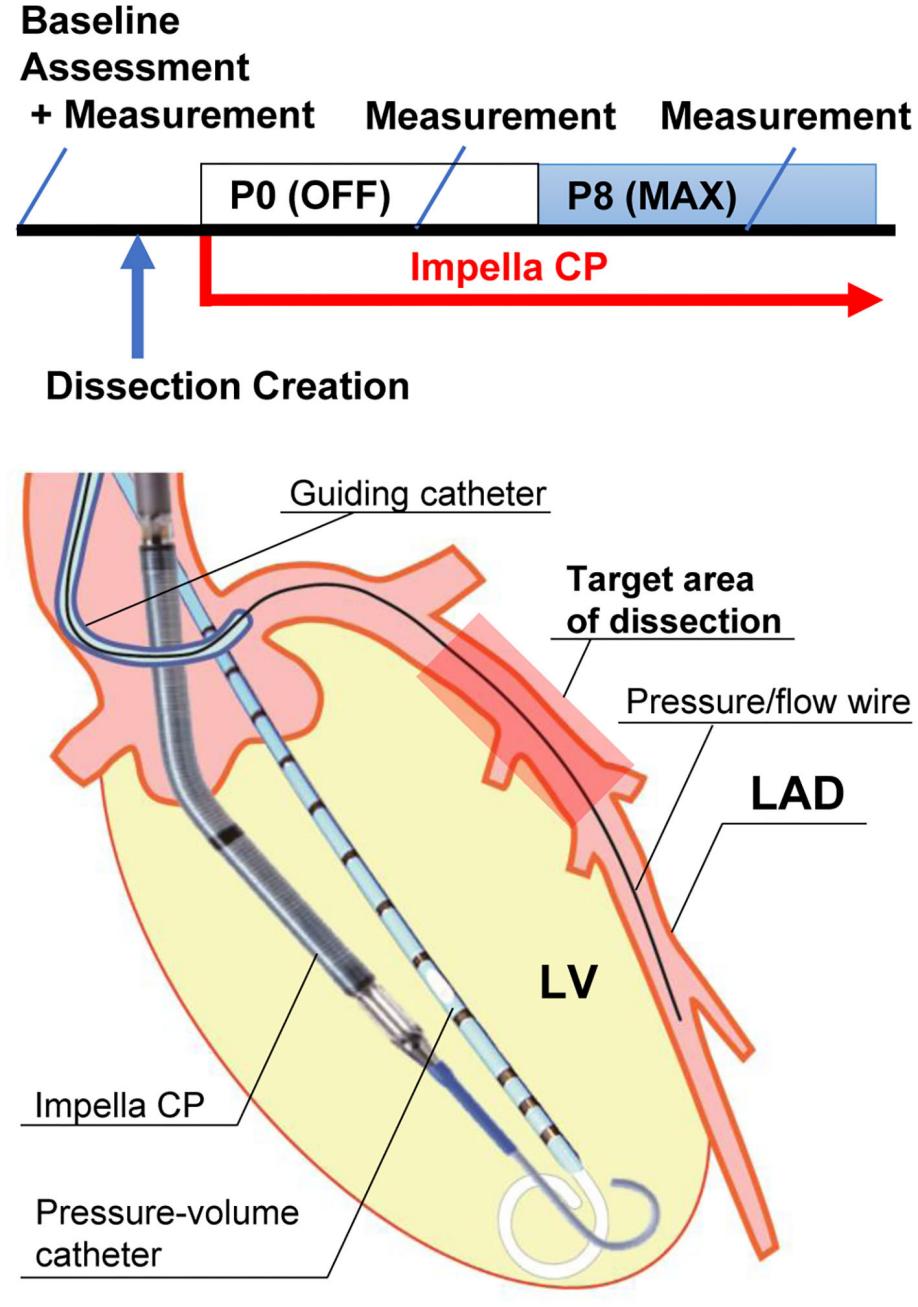
Experimental protocol and illustration of equipment. The upper panel shows the experimental protocol. “Baseline assessment” includes echocardiogram and pressure measurement. “Measurement” includes pressure–volume measurement, coronary pressure/flow, and coronary angiogram. The lower panel shows an illustration of the equipment. An Impella CP and a pressure–volume catheter are in the left ventricle (LV). A pressure/flow wire is placed in the mid-left anterior descending artery (LAD) distal to the dissection. The wire was aligned parallel to the LAD lumen for accurate measurement. Impella CP provided 2.3–3.0 L/min of flow from the LV to the aorta at P8 maximal (Max) flow.

Four female and four male Yorkshire pigs aged 4–5 months were premedicated using intramuscular tiletamine/zolazepam (Telazol, 8.0 mg/kg, Fort Dodge, IA). After placement of an intravenous line, animals were intubated and ventilated with 100% oxygen and kept in a supine position. General anesthesia was maintained by intravenous infusion of propofol (Diprivan, 10 mg/kg/h, Fresenius Kabi, USA) throughout the experiment. Six-lead electrocardiogram, rectal temperature, and pulse oximetry were monitored continuously and recorded at 5-min intervals. Transthoracic echocardiography was performed to exclude major structural heart disease. A 7-Fr Swan–Ganz catheter (Edwards Lifesciences, Irvine, CA) and a Ventri-Cath 510 conductance-catheter (Millar Instruments, Houston, TX) were inserted as described below in specific methods. Left atrial pressure was measured by a 5-Fr Hockey stick catheter (Cordis, Santa Clara, CA) placed via the interatrial septum. Animals underwent the induction of coronary dissection after baseline echocardiographic and pressure–volume (PV) measurements were made. ComboWire or a pair of pressure and flow wires (Volcano, San Diego, CA) were inserted into the left anterior descending artery (LAD) under fluoroscopic guidance, and the sensor was placed at the mid-LAD. After the CAD was created, Impella CP (Abiomed, Danvers, MA) was inserted into the LV. Flow was initially set to P0 (no support) and then increased to P8 (maximal pump flow) accompanied by LV and coronary hemodynamic measurements (Figure 1). At the end of the study, animals were euthanized, and the LAD was dissected for macroscopic examination. The protocol was approved by the Committee on Research Animal Care at Icahn School of Medicine at Mount Sinai.

### Echocardiography

A Philips iE33 ultrasound system (Philips Medical Systems, Andover, MA) was used to acquire echocardiographic data as described elsewhere (8). Complete Doppler transthoracic echocardiographic measurements were performed before CAD. Two-dimensional four-chamber images were acquired from the subxiphoid window, and cross-sectional images of the LV were obtained from the right intercostal spaces. LV volumes and ejection fraction were calculated using three-dimensional full-volume algorithms including a semiautomated border detection system. The endocardial detection accuracy was manually checked and optimized.

### Pressure and Volume Measurements

The methods for pressure measurement were previously described (9). Briefly, a Swan–Ganz catheter (Edwards Lifesciences, Irvine, CA) was advanced to the main pulmonary artery, and pressure data were collected. A bolus of ~0.25 × body weight (in kilograms) ml of cold saline was injected into the right atrium to obtain cardiac output by the thermodilution method. Through the peripheral arterial access, a 7-Fr, 12-electrode, dual-field conductance-catheter (Ventri-Cath 510, Millar Instruments, Houston, TX) was advanced to the LV to obtain hemodynamic parameters. All measurements were acquired after confirmation of hemodynamic stability for 3 min. Data analyses were performed using iox2 version 2.5.1.10 (Emka Technologies, Falls Church, VA). The conductance-catheter gain factor α was calculated as the ratio of cardiac output measured by thermodilution to that measured by the conductance-catheter. Parallel conductance was adjusted using the end-diastolic volume obtained from three-dimensional echocardiography. PV loops were visualized with Python 3.7.3.

### Creation of Coronary Dissection

A 0.018-in. stiff guidewire was advanced into the LAD using 7-Fr Hockey-stick coronary guiding catheter (Cordis) modified to a blunt-cut tip. In the first two animals (#1 and #2), coronary dissection was induced only by scratching the intima of the LAD using the stiff wire. In animals #3–#8, dissection was induced by deep engagement of the modified 7-Fr guiding catheter with (#3 and #4) and without wire injury (#5–#8). The modified guiding catheter was deeply engaged into the LAD, and its blunt-cut tip was wedged on the arterial wall. Half-wedged guiding blood pressure was obtained, and 7–9 ml of contrast was forcefully injected under fluoroscopic guidance until obvious coronary dissections developed. Wire injury was abbreviated in the last four animals because obvious dissection with a flap was reproducibly induced using guiding catheter method.

### Coronary Pressure and Flow Velocity Measurement and Signal Analysis

As described in *Study Protocol*, ComboWire or a pair of pressure and flow wires were placed at mid-LAD, which was distal to the LAD dissected lesion. Coronary pressure and flow velocity within the true lumen distal to the dissection were recorded and stored via iox2 at a sampling rate of 1 kHz. Python 3.7.3 was used for signal analyses. Acquired pressure and flow traces were down-sampled to 200 Hz. After noise reduction using a Savitzky–Golay filter (window = 11, polynomials = 3), each cardiac cycle was determined by the local minimum value of the pressure trace. Ten consecutive cycles of raw pressure and flow traces were ensemble-averaged. From these averaged waveforms, maximum, minimum, and mean coronary pressures and flow velocities were obtained.

### Angiography and Left Anterior Descending Artery Flow Delay Quantification

After pressure and flow measurements, the guiding catheter orientation was adjusted to inject contrast evenly into both the LAD and left circumflex artery (LCX). Coronary angiograms were taken at a frame rate of 30 frames/s. To quantify the flow delay in LAD, its delay time (DT) was defined as:

$$\begin{array}{l} \left(\text{DT}\right)=\left(\text{LAD contrast filling time}\right)-\left(\text{LCX contrast filling time}\right), \end{array}$$

based on the coronary angiogram.

### Statistics

Continuous variables are expressed as mean ± SD. A paired Student's *t*-test was used to compare the differences between two conditions (Impella support levels P0 and P8). Statistical analysis was performed with R (version 3.5.2). Graphs were created using GraphPad Prism (version 8.2.1) for Windows (GraphPad Software, La Jolla, CA).

## Results

### Creation of Coronary Dissection

**Figure 2:**
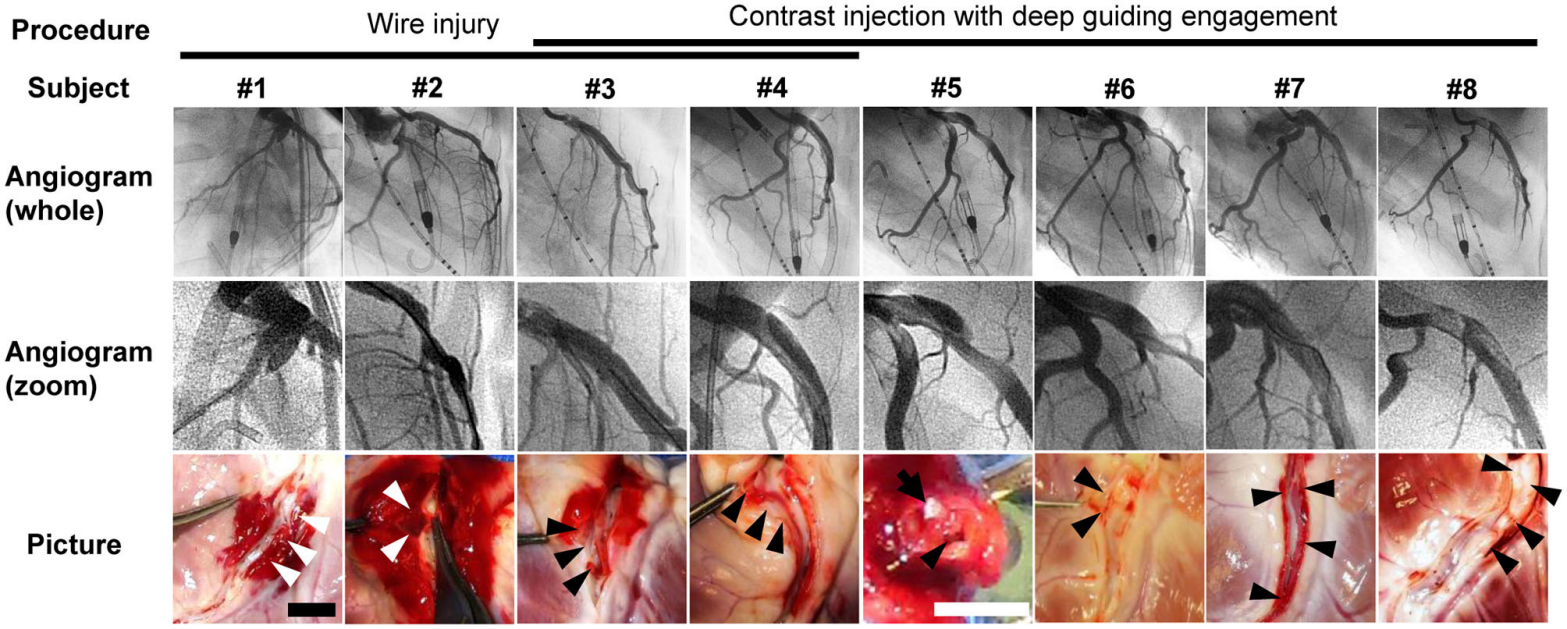
Angiograms and postmortem pictures of all animals. Procedure for creation of coronary dissection is indicated on the top. Left coronary angiograms were taken after the creation of coronary dissection of the left anterior descending artery (LAD). Angiogram #1 was taken from the left anterior oblique 30° view. Angiograms #2–#8 were from the right anterior oblique 90° view. Zoomed angiograms show coronary dissections in LAD. The bottom images are postmortem pictures of the LAD cut open longitudinally (#1–#4 and #6–#8) or cross-sectionally (#5). Arrowheads (white and black) indicate dissected lumen. White arrowheads indicate relatively limited dissections with periarterial hematoma. Black arrowheads indicate dissections with smooth internal surfaces and surrounding hematoma. No evident re-entry was seen in any of the animals. In Picture #5, a 24-gauge cannula (a white tip) is placed in the true lumen (black arrow). Scale bars of the pictures are 10 mm (black, #1–#4 and #6–#8) and 5 mm (white, #5). LCA, left coronary artery.

All eight animals survived the coronary dissection procedure to the completion of each study. Baseline echocardiographic and LV hemodynamics data before CAD creation were within normal ranges (Table 1). Coronary dissection in the proximal LAD was successfully created in all eight animals. The induction method and results of the dissection for each animal are summarized in Figure 2. The wire-injury method produced only small dissections with periarterial hematomas (animals #1 and #2; white arrowheads, Figure 2). Deep engagement of the blunt-cut tip guiding catheter with forceful contrast injection was the most successful method in creating dissections with a large flap (animals #3–#8; black arrowheads, Figure 2). In these animals, a flap with a contrast-filled false lumen was seen in the proximal portion of the LAD (Figure 2). One animal (animal #7) exhibited a spiral dissection with the false lumen extending to the distal LAD. After dissection, one animal (animal #8) exhibited thrombolysis in myocardial infarction (TIMI)-1 grade flow (DT 1.07 s), while the other seven animals showed TIMI-2 or 3 flow (DT 0.26 ± 0.16 s). Postmortem gross examination revealed dissections with smooth internal surfaces with surrounding hematoma. No evident re-entry was seen.

**Table 1:** Baseline characteristics of experimental pigs.

BW, kg	51.0 ± 5.5
**2D echocardiography**	
LV mass, g	134.1 ± 22.6
LA diameter, mm	47.1 ± 3.3
**3D echocardiography**	
LVEF, %	69.9 ± 5.9
LVESV, ml	100.4 ± 21.0
LVEDV, ml	30.3 ± 8.2
**Invasive measurements**	
Heart rate, bpm	58.4 ± 6.1
Cardiac output, L/min	3.46 ± 0.54
Systolic arterial pressure, mmHg	131.9 ± 21.8
Diastolic arterial pressure, mmHg	78.4 ± 17.1
Mean arterial pressure, mmHg	98.5 ± 20.0
Mean pulmonary pressure, mmHg	14.0 ± 2.3
LVESP, mmHg	114.6 ± 22.9
LVEDP, mmHg	10.8 ± 4.6
RV systolic pressure, mmHg	27.3 ± 5.2
RV mean pressure, mmHg	4.0 ± 2.7
Mean pulmonary wedge pressure, mmHg	5.8 ± 2.0
Mean LA pressure, mmHg	5.8 ± 2.1
Mean right atrial pressure, mmHg	2.6 ± 1.3

*LV, left ventricle; LA, left atrium; LVED, left ventricular ejection fraction; LVESV, left ventricular end-systolic volume; LVEDV, left ventricular end-diastolic volume; LVESP, left ventricular end-systolic pressure; LVEDP, left ventricular end-diastolic pressure; RV, right ventricle*.

### Impact of Impella on Coronary True-Lumen Hemodynamics Distal to Dissection

**Figure 3:**
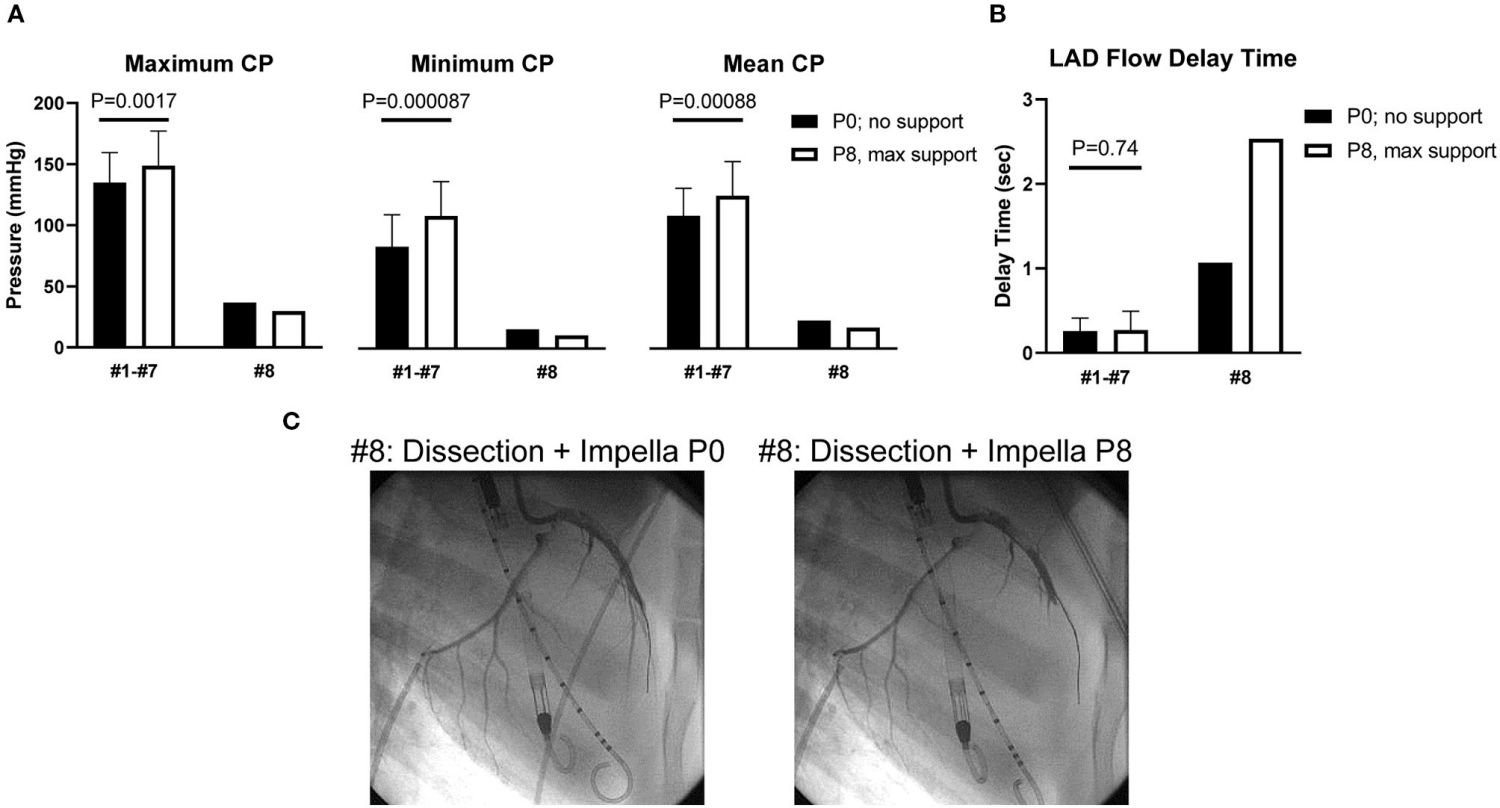
Coronary pressure and flow of the dissected left anterior descending artery. **(A)** Coronary pressures (CPs) of the true lumen. Minimum and mean coronary pressures increased at Impella max flow (P8) in animals #1–#7, whereas they decreased in animal #8. **(B)** Left anterior descending artery (LAD) flow delay time (DT) was defined as (LAD filling time) – (LCX filling time). Animal #8 showed increased DT after dissection, and DT prolonged under maximum Impella support, while animals #1–#7 showed unchanged DT. **(C)** Coronary angiograms of animal #8. Coronary dissection was made in proximal LAD. LAD contrast delay was prominent at maximal Impella support (P8, right side). Movies of the angiograms are provided in Supplementary Movies.

**Figure 4:**
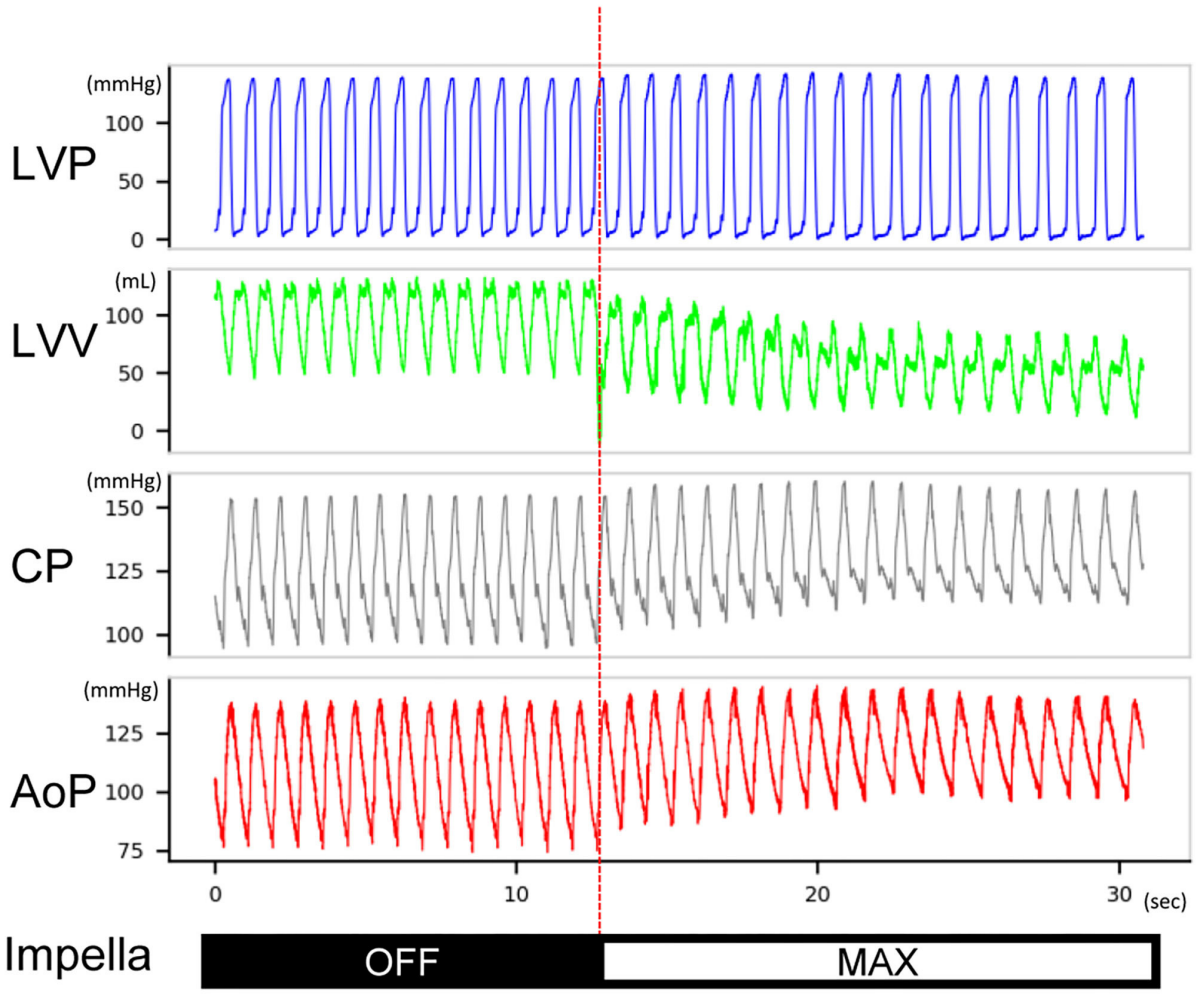
Typical hemodynamic traces of Impella LV support on a heart with non-flow-limiting coronary dissection. After Impella was turned on, the left ventricular volume decreased, the coronary mean pressure increased, and the diastolic aortic pressure increased. The length of the traces is 30 s. This record was from animal #5. LVP, left ventricular pressure; LVV, left ventricular volume; CP, coronary pressure; AoP, aortic pressure.

The Impella console indicated an average mean flow of 2.7 ± 0.2 L/min at the maximum P8 support setting. Coronary pressures distal to the dissection increased in animals #1–#7 with maximum Impella support (maximum 134.9 ± 24.7–148.6 ± 28.6 mmHg, *P* = 0.0017; minimum 83.1 ± 26.3–108.2 ± 28.3 mmHg, *P* < 0.001; mean 108.4 ± 22.5–124.7 ± 28.0 mmHg, *P* < 0.001, Figure 3A). In these animals, coronary pressure increased along with aortic pressure after switching on the Impella pump (Figure 4). Meanwhile, LAD true-lumen pressure distal to the dissection decreased in animal #8 (maximum pressure 36.7–29.6 mmHg, minimum pressure 15.8–10.8 mmHg, and mean pressure 22.9–17.1 mmHg). This was accompanied by a further deterioration of coronary flow as evaluated by coronary angiogram. In this animal, maximal Impella support resulted in further delay of LAD flow (DT 1.07 to 2.53 s, Supplementary Movies 1, 2), whereas the DT remained similar in the other seven animals (DT 0.26 ± 0.16–0.27 ± 0.22 s, *P* = 0.74, Figures 3B,C). In all animals, maximum and mean coronary flow velocities tended to decrease after Impella LV support (maximum 121.24 ± 71.03–84.31 ± 34.24 cm/s, *P* = 0.074; mean 63.50 ± 28.66–48.31 ± 13.30 cm/s, *P* = 0.17, Supplementary Figure 1).

### Impella Support Unloads Left Ventricular in Coronary Artery Dissection Setting

Changes in LV hemodynamics before and after Impella support were examined in the six animals with a large flap (animals #3–#8). LV Impella support at P8 (maximum support) vs. P0 (no support) demonstrated a significant decrease in the LV end-diastolic pressure [20.6 ± 6.6 (P0) vs. 12.0 ± 3.4 (P8) mmHg, *P* = 0.032], the LV end-diastolic volume (127 ± 32 vs. 97 ± 26 ml, *P* = 0.015), stroke volume (68 ± 16 vs. 48 ± 14 ml, *P* = 0.003), stroke work (5,744 ± 1,866 vs. 4,424 ± 1,650 mmHg·ml, *P* = 0.003), and heart rate (71.4 ± 6.6 vs. 64.9 ± 9.3/min, *P* = 0.014) after Impella support. Total cardiac output (sum of native and Impella flow) increased significantly (4.78 ± 0.83 vs. 5.70 ± 0.46 L/min, *P* = 0.03) (Table 2). These results indicate that Impella unloads the LV in the presence of LAD dissection and decreases ventricular work while augmenting the systemic perfusion. Notably, the TIMI-1 animal exhibited a different pattern in the shifting of PV loop after CAD and Impella as compared with the rest of the animals. In the TIMI-1 animal (animal #8), PV loop shifted right-downward after CAD creation and end-systolic volume increased after Impella support despite reduced stroke work, whereas in other animals (animals #3–#7), PV loops shifted leftward on Impella, which is a typical LV unloading pattern (Supplementary Figure 2). The unique PV loop shifting pattern in the TIMI-1 animal suggests potential influence of myocardial ischemia after coronary dissection and Impella support.

**Table 2:** Effect of Impella on LV parameters after coronary dissection in animals with large coronary flap.

	**Impella off**	**Impella max flow**	***P*-value**
	**(*N* = 6)**	**(*N* = 6)**	
LV end-diastolic pressure, mmHg	20.6 ± 6.6	12.0 ± 3.4	0.032
LV end-systolic pressure, mmHg	112.2 ± 28.6	126.9 ± 22.9	0.066
LV end-diastolic volume, ml	127 ± 32	97 ± 26	0.015
LV end-systolic volume, ml	71 ± 32	63 ± 27	0.14
Stroke volume, ml	68 ± 16	48 ± 14	0.003
Stroke work, mmHg·ml	5,744 ± 1,866	4,424 ± 1,650	0.003
Heart rate, bpm	71.4 ± 6.6	64.9 ± 9.3	0.014
Cardiac output, L/min	4.78 ± 0.83	5.70 ± 0.46	0.03

*Cardiac output is a summation of one from left ventricular contraction and one from Impella*.

*LV, left ventricle*.

### Impact of Impella on Coronary False-Lumen Pressure in Animal #8

**Figure 5:**
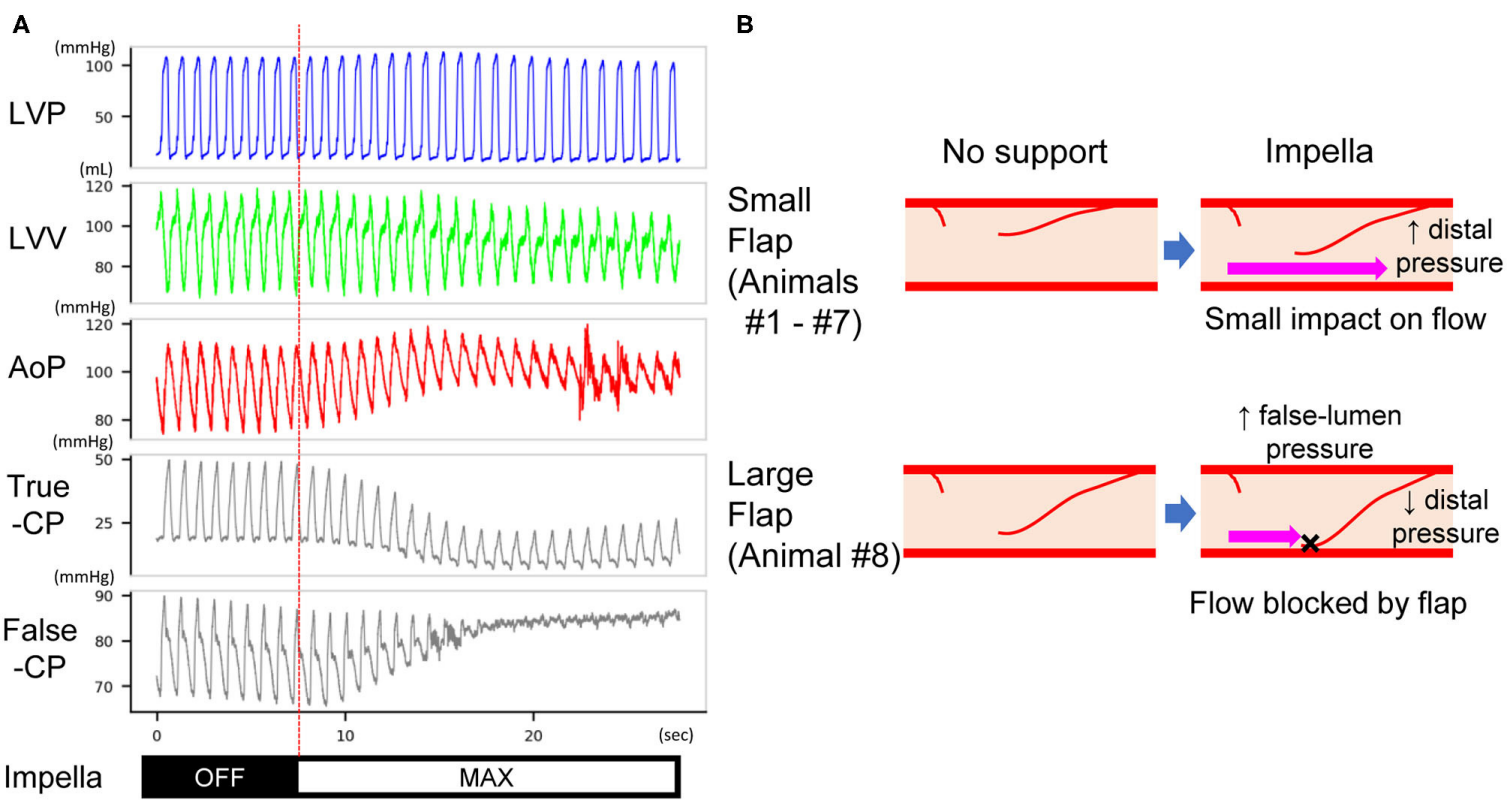
**(A)** Pressure traces of the LV, true and false lumens of dissected coronary artery in animal #8. After Impella was turned on, the mean pressure of the true lumen decreased, whereas that of the false lumen increased. The length of the traces is 30 s. LVP, left ventricular pressure; LVV, left ventricular volume; AoP, aortic pressure; True-CP, pressure of coronary true lumen; False-CP, pressure of coronary false lumen. **(B)** Expected mechanism of impaired distal (true-lumen) flow at Impella P8. A small flap does not affect the true-lumen flow under maximal Impella support (upper panels). A large flap increases the false-lumen pressure and blocks coronary flow under maximal Impella support (lower panels). This results in a decrease of true-lumen pressure as shown in **(A)**.

After measurement of coronary pressure in the true lumen, the pressure wire was able to be inserted into the false lumen in animal #8, which had a deterioration of distal coronary flow. The mean pressure in the false lumen increased at P8 support in this animal (74.2–99.0 mmHg), coinciding with increased aortic pressure, whereas the true-lumen pressure distal to the dissection decreased (22.9–17.1 mmHg) (Figure 5A). This was accompanied by a delay in antegrade flow assessed by coronary angiogram (Figure 3C).

## Discussion

In this study, we developed a novel animal model of CAD that will allow for preclinical studies of CAD, a rare but serious condition. To our knowledge, this is the first description of a large animal model of CAD both with and without coronary flow limitation. This model will enable future investigations into CAD and testing of new therapeutic approaches. Moreover, using this new model, we demonstrated that Impella is safe and effective in unloading the LV in the majority of the CAD cases, but it might pose a potential negative impact on coronary flow when there is a large intimal flap.

### Novel Pig Model of Coronary Artery Dissection

Different types of CAD could be induced without any mortality in pigs. Two (animals #1 and #2) developed small CAD with periarterial hematoma by the wire injury method. In contrast, six animals exhibited clear contrast-filled false lumen (animals #3–#8). Thus, deep engagement of a blunt-cut tip guiding catheter with forceful contrast injections was the most successful method for creating a large dissection with a flap. Catheter modification, deep engagement of the catheter into the LAD with half-wedged pressure, and forceful contrast injections were the keys to successfully induce large intimal tear and a flap. In one animal (#8), a flow-limiting dissection was induced by generating an extensive intimal tear. In this animal, flow in the true lumen was further deteriorated by maximal Impella support. Although we attempted to induce similar flow limiting dissection in other animals, this was not successful. Therefore, although coronary dissection with a large intimal tear and flap can be reproducibly induced, the difficulty in controlling the severity of dissection may represent the limitation of the model.

Both types of dissections are clinically relevant (10). Two clinically important occasions are iatrogenic CAD and SCAD. Iatrogenic coronary dissection is a rare but life-threatening complication of PCI (2, 11). Its presence is associated with high in-hospital mortality (2). Selection of the true lumen during PCI in CAD cases is sometimes difficult, and intervention failure is higher than in other forms of myocardial infarction (12). An established management strategy for iatrogenic CAD is lacking. Currently, conservative medical approaches, PCI, CABG, and even cardiac transplantation have been utilized to treat the pathology. However, PCI is the most often deployed therapeutic approach as it is the quickest way to restore coronary blood flow to reduce ischemic damage and stabilize patient hemodynamics, if successful (1, 13, 14). The lack of consensus on how best to treat iatrogenic CAD emphasizes the need for more basic research.

Treatment options for dissection are further complicated in the setting of SCAD. For reasons still unclear, patients with SCAD present with uncharacteristically friable arteries. Upon dissection, treating a SCAD patient with PCI is difficult, as any physical contact with the vessel wall can extend the dissection and increase mortality (15, 16). SCAD is being increasingly recognized since it affects relatively young females (<50 years of age) with few coronary risk factors (17). It is the most common cause of pregnancy-associated myocardial infarction (18) with significantly higher mortality compared with atherosclerotic myocardial infarction, a 50% failure rate of PCI, and 2.5% fetal mortality (19). As with CAD, there is no consensus on how to best treat SCAD patients. While clinical experience is limited, conservative management is often the chosen course of action as it limits potential further iatrogenic coronary damage; many of these dissections heal on their own within weeks if the patient can be bridged through the initial insult (3, 20). These patients can suffer from cardiac dysfunction and hemodynamic collapse due to the myocardial infarction if flow is not restored or the ischemic burden is not relieved.

For these reasons, new devices or management strategies are being sought to reduce myocardial injury associated with CAD. To this end, our model offers a new experimental platform for studying the pathophysiology of CAD and for the development of new therapeutic measures. Our data demonstrate the utility of the model for future investigations into CAD. Our results also suggest that Impella CP unloads the LV in the setting of CAD without propagation of the dissection, stabilizes hemodynamics, and increases coronary pressure distal to the dissection in majority of cases.

### Impella Use in Coronary Dissection With Hemodynamic Instability

Coronary dissection is associated with a greater risk of developing cardiogenic shock than atherosclerosis-related ST-elevated myocardial infarction (STEMI) and is thus more likely to require mechanical circulatory support such as Impella. In a recent study of patients with SCAD-associated STEMI, more than half (53%) had a low initial TIMI flow grade, and cardiogenic shock was present more frequently (19 vs. 9%) than in atherosclerosis-associated STEMI (21). An analysis of pregnancy-associated SCADs revealed that 24% exhibited cardiogenic shock and 28% required mechanical support (19). Moreover, male and female patients with iatrogenic catheter-induced CADs are more likely to develop cardiogenic shock than other patients undergoing PCI (7.9 vs. 1.9%), with the former exhibiting flow limitation in 23% of cases (2).

Among the available mechanical circulatory assist device options in the clinic, Impella offers rapid insertion in the catheterization laboratory and provides more physiological circulation than does extracorporeal membrane oxygenation. Although these features render Impella the primary option for CAD cases accompanied by cardiogenic shock, its impact on coronary flow and LV unloading remains unknown. Using our newly established porcine model, we studied the impact of Impella on both coronary hemodynamics and LV. Impella support in our study provided partial LV support at the maximal support level of the machine. Consistent with previous reports (22–24), Impella effectively unloaded LV and increased aortic pressure (Figures 4, 5A). However, coronary arterial pressure distal to the dissection failed to increase in one animal (#8), whereas the false lumen pressure increased (Figure 5A). This was accompanied by decreased coronary flow as evaluated by the coronary angiogram. This pig was the only animal that exhibited significant delay in coronary flow before Impella support, and the other animals had TIMI-2 or 3 despite a major coronary dissection with a flap. Interestingly, a coronary pressure wire inserted into the false lumen registered an increase in pressure when Impella was turned on, in contrast to decreased pressure in the true lumen. The proposed mechanism for the deterioration of coronary flow that we observed is illustrated in Figure 5B. Impella CP increases mean aortic pressure by increasing the forward flow (Figure 4). If the intimal flap is small, changes in aortic and proximal coronary pressures do not affect distal coronary flow and pressure (Figure 5B, upper panel). Instead, if the flap is large, increased aortic pressure by Impella can increase pressure in the false lumen and displace the flap, which can obstruct antegrade flow into the true lumen (Figure 5B, lower panel). This notion was supported by the observed pressure increase in the false lumen and decrease in the true lumen at maximum Impella support in animal #8 (Figure 5A). PV loop analysis suggested potential influence of ischemia in flow-limiting CAD (Supplementary Figure 2), although further study is needed to confirm this. Additionally, although subjective, we noted that pressure wire re-placement into the true lumen in animal #8 progressed much more easily when Impella was turned off. Therefore, our results suggest that the development of further ischemia should be monitored during Impella use in CAD patients with a large flap. Particularly in flow-limiting (i.e., TIMI-1) CAD patients, careful evaluation of ischemia should be performed by changing the Impella flow. For PCI in these patients, temporarily decreasing the Impella flow could facilitate wire placement into the true lumen.

Impella has been shown to increase coronary flow to the infarcted area after myocardial infarction (7). However, in our study, the flow wire data exhibited decreases in coronary flow velocity in all animals, despite increased coronary pressure. Increased coronary pressure with decreased coronary flow suggests that there is an increase in coronary resistance. This is likely due to coronary autoregulation in which the flow is autoregulated to maintain adequate perfusion to the myocardium in response to decreased myocardial oxygen consumption. Indeed, in our previous study, we found that non-infarcted remote myocardial tissue perfusion showed variable reactions against Impella LV support (7). We are currently studying the impact of LV unloading on coronary hemodynamics and autoregulation in the non-ischemic myocardium, and we hope to verify our assumptions in the near future.

### Limitation

Our model lacks the important characteristic of friable vessels observed in SCAD patients. Currently, the underlying mechanism of SCAD pathology is unknown and cannot be accurately recreated in animal. However, our model was useful in testing the hypothesis that Impella LV support can stabilize the SCAD or the other types of CAD. In our study, the Impella was inserted in animals with CAD, but not in those with cardiogenic shock or obvious ongoing ischemia. While our results support successful unloading of the LV, actual efficacy in the setting of cardiogenic shock needs to be examined in future studies. We did not evaluate the extent of myocardial ischemic injury or systemic hemodynamic decline after CAD creation. Because we did not quantify the impact of observed coronary flow deterioration on LV and systemic hemodynamics, we cannot make definite statements regarding the significance of the prevention or management of flow deterioration by Impella in myocardial salvage or the prognosis for patients.

## Conclusion

We successfully created a novel porcine model of CAD. This model will enable preclinical studies targeting CAD in human-sized animals and might offer new insights. This is the first study to investigate how a temporary mechanical circulatory support device may be useful in the setting of CAD.

## Data Availability Statement

All datasets generated for this study are included in the article/Supplementary Material.

## Ethics Statement

The animal study was reviewed and approved by Committee on Research Animal Care at Icahn School of Medicine at Mount Sinai.

## Author Contributions

TK and KI conceived, designed the experiments, and analyzed the data. TK, KY, OB, ST, SM, and KI performed the experiments. TK, KY, and KI wrote the manuscript. All authors critically revised and approved the final manuscript.

## Conflict of Interest

TK has received a fellowship grant from Abiomed. KI serves as a PI of a research grant to the institution from Abiomed Inc. The remaining authors declare that the research was conducted in the absence of any commercial or financial relationships that could be construed as a potential conflict of interest.

## Supplementary Material

Supplementary Figure 1 Coronary flow velocity of the true lumen of the dissected coronary artery. In animals #1–#7, maximum and mean coronary flow velocity of the true lumen of the dissected left anterior descending artery tended to decrease at Impella maximum flow (P8).

Supplementary Figure 2 Representative left ventricular pressure-volume loops at baseline, after CAD and Impella. **(A)** Thrombolysis in Myocardial Infarction (TIMI)-3 animal (#4). After Impella support, the pressure volume (PV) loop shifted left-downward (dash-dot line, red). Each loop is an ensemble-average of five consecutive stable cardiac cycles of each stage. **(B)** Animal (#8) with TIMI-1 flow after coronary arterial dissection (CAD). The PV loop shifted right-downward after CAD induction (dash line, green), compared with the baseline loop (solid line, blue). After Impella support, end-systolic volume increased despite decreased left ventricular stroke work (dash-dot line, red). Lack of left-downward shift of PV loops in this animal suggests potential influence of myocardial ischemia after CAD creation and Impella initiation. Each loop is an ensemble-average of five consecutive stable cardiac cycles of each stage. CAD, coronary arterial dissection.

Supplementary Movie 1 The left coronary angiogram of animal #8 without Impella support. The right anterior oblique (RAO) 90° view. A picture is provided in the left panel of Figure 3C. A dissected proximal portion of the left anterior descending (LAD) artery is seen.

Supplementary Movie 2 The left coronary angiogram of animal #8 with maximal Impella support. The right anterior oblique (RAO) 90° view. A picture is provided in the right panel of Figure 3C. A dissected proximal portion of the left anterior descending (LAD) artery is seen. Delayed contrast run-off in the LAD is noted compared to no Impella support (Supplementary Movie 1).

## References

[1] Eshtehardi P Adorjan P Togni M Tevaearai H Vogel R Seiler C. Iatrogenic left main coronary artery dissection: incidence, classification, management, and long-term follow-up. Am Heart J. (2010) 159:1147–53. 10.1016/j.ahj.2010.03.012 20569732

[2] Hiraide T Sawano M Shiraishi Y Ueda I Numasawa Y Noma S. Impact of catheter-induced iatrogenic coronary artery dissection with or without postprocedural flow impairment: a report from a Japanese multicenter percutaneous coronary intervention registry. PLoS ONE. (2018) 13:e0204333. 10.1371/journal.pone.0204333 30265698 PMC6162084

[3] Hayes SN Kim ESH Saw J Adlam D Arslanian-Engoren C Economy KE. Spontaneous coronary artery dissection: current state of the science: a scientific statement from the American heart association. Circulation. (2018) 137:e523–57. 10.1161/CIR.0000000000000564 29472380 PMC5957087

[4] Strom JB Zhao YS Shen CY Chung M Pinto DS Popma JJ. National trends, predictors of use, and in-hospital outcomes in mechanical circulatory support for cardiogenic shock. Eurointervention. (2018) 13:E2152–9. 10.4244/Eij-D-17-00947 29400657

[5] Doshi R Patel K Decter D Gupta R Meraj P. Trends in the utilisation and in-hospital mortality associated with short-term mechanical circulatory support for heart failure with reduced ejection fraction. Heart Lung Circ. (2019) 28:e47–50. 10.1016/j.hlc.2018.03.025 29705384

[6] Burkhoff D Sayer G Doshi D Uriel N. Hemodynamics of mechanical circulatory support. J Am Coll Cardiol. (2015) 66:2664–74. 10.1016/j.jacc.2015.10.017 26670067

[7] Watanabe S Fish K Kovacic JC Bikou O Leonardson L Nomoto K. Left ventricular unloading using an impella CP improves coronary flow and infarct zone perfusion in ischemic heart failure. J Am Heart Assoc. (2018) 7:e006462. 10.1161/JAHA.117.006462 29514806 PMC5907535

[8] Ishikawa K Chemaly ER Tilemann L Fish K Ladage D Aguero J. Assessing left ventricular systolic dysfunction after myocardial infarction: are ejection fraction and dP/dt(max) complementary or redundant? Am J Physiol Heart Circ Physiol. (2012) 302:H1423–8. 10.1152/ajpheart.01211.2011 22307667 PMC3330783

[9] Ishikawa K Aguero J Oh JG Hammoudi N Fish LA Leonardson L. Increased stiffness is the major early abnormality in a pig model of severe aortic stenosis and predisposes to congestive heart failure in the absence of systolic dysfunction. J Am Heart Assoc. (2015) 4:e001925. 10.1161/JAHA.115.001925 25994443 PMC4599422

[10] Huber MS Mooney JF Madison J Mooney MR. Use of a morphologic classification to predict clinical outcome after dissection from coronary angioplasty. Am J Cardiol. (1991) 68:467–71. 10.1016/0002-9149(91)90780-o 1872273

[11] Venkitachalam L Kip KE Selzer F Wilensky RL Slater J Mulukutla SR. Twenty-year evolution of percutaneous coronary intervention and its impact on clinical outcomes: a report from the national heart, lung, and blood institute-sponsored, multicenter 1985-1986 PTCA and 1997-2006 dynamic registries. Circ Cardiovasc Interv. (2009) 2:6–13. 10.1161/CIRCINTERVENTIONS.108.825323 20031687 PMC3024012

[12] Tweet MS Eleid MF Best PJ Lennon RJ Lerman A Rihal CS. Spontaneous coronary artery dissection: revascularization versus conservative therapy. Circ Cardiovasc Interv. (2014) 7:777–86. 10.1161/CIRCINTERVENTIONS.114.001659 25406203

[13] Cappelletti A Margonato A Rosano G Mailhac A Veglia F Colombo A. Short- and long-term evolution of unstented nonocclusive coronary dissection after coronary angioplasty. J Am Coll Cardiol. (1999) 34:1484–8. 10.1016/s0735-1097(99)00395-2 10551696

[14] Hayman S Lavi S. Healing of iatrogenic coronary dissection and intramural hematoma: insights from OCT. J Invasive Cardiol. (2018) 30:E12–3. 29289954

[15] Saw J Aymong E Sedlak T Buller CE Starovoytov A Ricci D. Spontaneous coronary artery dissection: association with predisposing arteriopathies and precipitating stressors and cardiovascular outcomes. Circ Cardiovasc Interv. (2014) 7:645–55. 10.1161/CIRCINTERVENTIONS.114.001760 25294399

[16] Saw J Humphries K Aymong E Sedlak T Prakash R Starovoytov A. Spontaneous coronary artery dissection: clinical outcomes and risk of recurrence. J Am Coll Cardiol. (2017) 70:1148–58. 10.1016/j.jacc.2017.06.053 28838364

[17] Sharma S Kaadan MI Duran JM Ponzini F Mishra S Tsiaras SV. Risk factors, imaging findings, and sex differences in spontaneous coronary artery dissection. Am J Cardiol. (2019) 123:1783–7. 10.1016/j.amjcard.2019.02.040 30929769

[18] Elkayam U Jalnapurkar S Barakkat MN Khatri N Kealey AJ Mehra A. Pregnancy-associated acute myocardial infarction: a review of contemporary experience in 150 cases between 2006 and 2011. Circulation. (2014) 129:1695–702. 10.1161/CIRCULATIONAHA.113.002054 24753549

[19] Havakuk O Goland S Mehra A Elkayam U. Pregnancy and the risk of spontaneous coronary artery dissection: an analysis of 120 contemporary cases. Circ Cardiovasc Interv. (2017) 10:e004941. 10.1161/CIRCINTERVENTIONS.117.004941 28302642

[20] Adlam D Alfonso F Maas A Vrints C Writing C. European society of cardiology, acute cardiovascular care association, SCAD study group: a position paper on spontaneous coronary artery dissection. Eur Heart J. (2018) 39:3353–68. 10.1093/eurheartj/ehy080 29481627 PMC6148526

[21] Lobo AS Cantu SM Sharkey SW Grey EZ Storey K Witt D. Revascularization in patients with spontaneous coronary artery dissection and ST-segment elevation myocardial infarction. J Am Coll Cardiol. (2019) 74:1290–300. 10.1016/j.jacc.2019.06.065 31488265

[22] Remmelink M Sjauw KD Henriques JP de Winter RJ Koch KT van der Schaaf RJ. Effects of left ventricular unloading by Impella recover LP2.5 on coronary hemodynamics. Catheter Cardiovasc Interv. (2007) 70:532–7. 10.1002/ccd.21160 17896398

[23] Raess DH Weber DM. Impella 2.5. J Cardiovasc Transl Res. (2009) 2:168–72. 10.1007/s12265-009-9099-4 20559984

[24] Alqarqaz M Basir M Alaswad K O'Neill W. Effects of impella on coronary perfusion in patients with critical coronary artery stenosis. Circ Cardiovasc Interv. (2018) 11:e005870. 10.1161/CIRCINTERVENTIONS.117.005870 29643128

