# Corrigendum: Novel Porcine Model of Coronary Dissection Reveals the Impact of Impella on Dissected Coronary Arterial Hemodynamics

**DOI:** 10.3389/fcvm.2021.646675

**Published:** 2021-04-08

**Authors:** Taro Kariya, Kelly P. Yamada, Olympia Bikou, Serena Tharakan, Satoshi Miyashita, Kiyotake Ishikawa

**Affiliations:** Cardiovascular Research Center, Icahn School of Medicine at Mount Sinai, New York, NY, United States

**Keywords:** coronary arterial dissection, spontaneous coronary artery dissection, impella, large animal, LV unloading, coronary angiography, intimal flap

In the original article, there was a mistake in Table 2, as published. The correct heart rate values appear below.

**Table 2:** Effect of Impella on LV parameters after coronary dissection in animals with large coronary flap.

	**Impella off**	**Impella max flow**	***P*-value**
	**(*N* = 6)**	**(*N* = 6)**	
LV end-diastolic pressure, mmHg	20.6 ± 6.6	12.0 ± 3.4	0.032
LV end-systolic pressure, mmHg	112.2 ± 28.6	126.9 ± 22.9	0.066
LV end-diastolic volume, ml	127 ± 32	97 ± 26	0.015
LV end-systolic volume, ml	71 ± 32	63 ± 27	0.14
Stroke volume, ml	68 ± 16	48 ± 14	0.003
Stroke work, mmHg·ml	5,744 ± 1,866	4,424 ± 1,650	0.003
Heart rate, bpm	71.4 ± 6.6	64.9 ± 9.3	0.014
Cardiac output, L/min	4.78 ± 0.83	5.70 ± 0.46	0.03

*Cardiac output is a summation of one from left ventricular contraction and one from Impella*.

*LV, left ventricle*.

The authors apologize for this error and state that this does not change the scientific conclusions of the article in any way. The original article has been updated.

