# Supplementary figures and images for: Novel Porcine Model of Coronary Dissection Reveals the Impact of Impella on Dissected Coronary Arterial Hemodynamics
Source: Front Cardiovasc Med. 2020 Sep 15;7:162. doi: 10.3389/fcvm.2020.00162 (PMC7522595; doi:10.3389/fcvm.2020.00162)

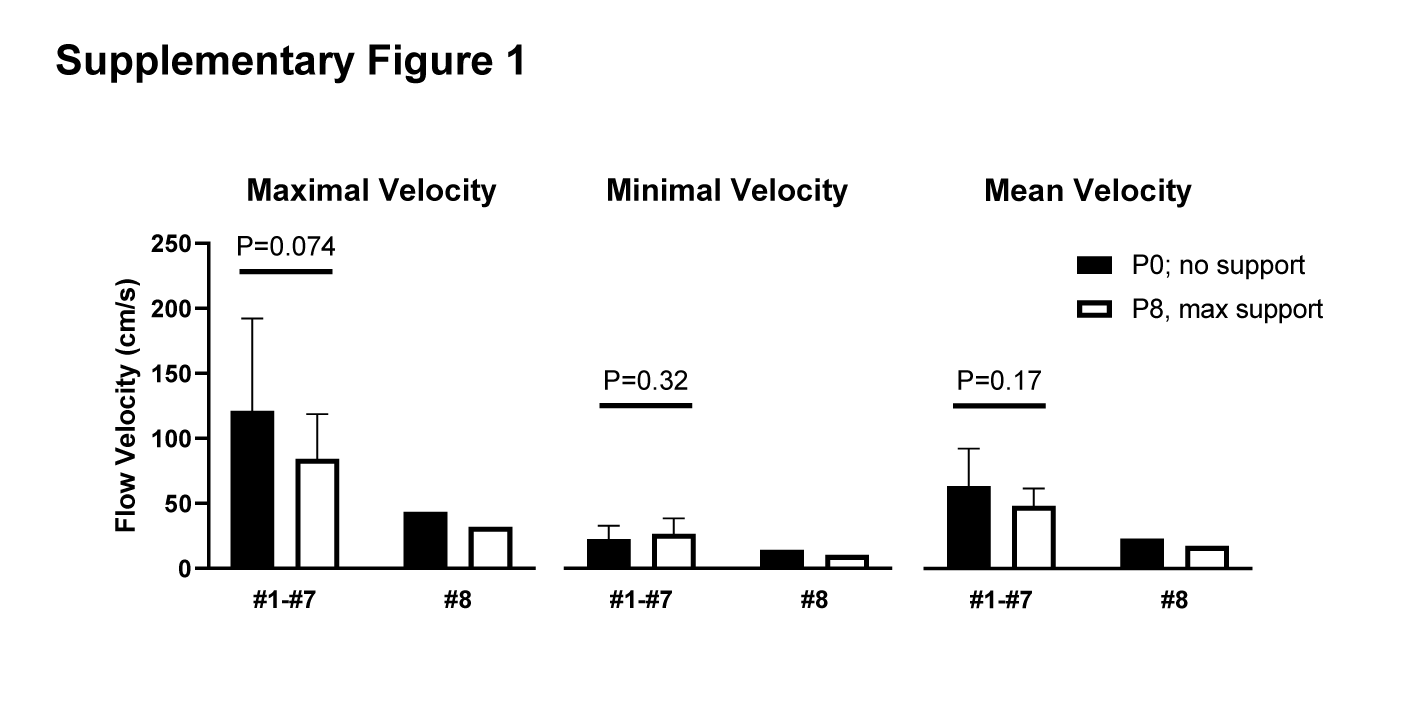

Supplement: Supplementary Figure 1 — Coronary flow velocity of the true lumen of the dissected coronary artery. In animals #1–#7, maximum and mean coronary flow velocity of the true lumen of the dissected left anterior descending artery tended to decrease at Impella maximum flow (P8). [file Image_1.TIF]

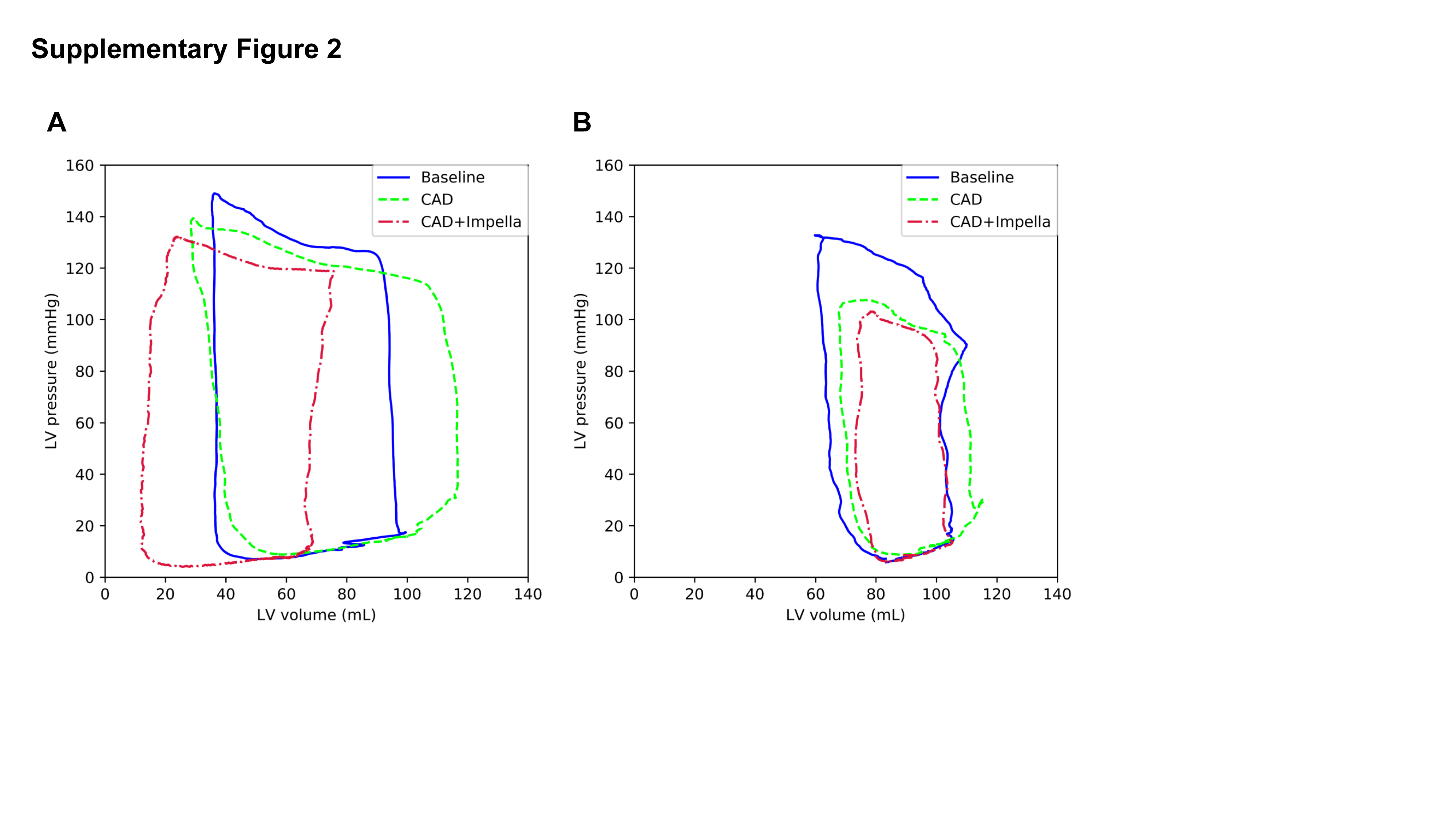

Supplement: Supplementary Figure 2 — Representative left ventricular pressure-volume loops at baseline, after CAD and Impella. (A) Thrombolysis in Myocardial Infarction (TIMI)-3 animal (#4). After Impella support, the pressure volume (PV) loop shifted left-downward (dash-dot line, red). Each loop is an ensemble-average of five consecutive stable cardiac cycles of each stage. (B) Animal (#8) with TIMI-1 flow after coronary arterial dissection (CAD). The PV loop shifted right-downward after CAD induction (dash line, green), compared with the baseline loop (solid line, blue). After Impella support, end-systolic volume increased despite decreased left ventricular stroke work (dash-dot line, red). Lack of left-downward shift of PV loops in this animal suggests potential influence of myocardial ischemia after CAD creation and Impella initiation. Each loop is an ensemble-average of five consecutive stable cardiac cycles of each stage. CAD, coronary arterial dissection. [file Image_2.TIF]
